# Efficacy, Safety, and Economics of Intravenous Levetiracetam for Status Epilepticus: A Systematic Review and Meta-Analysis

**DOI:** 10.3389/fphar.2020.00751

**Published:** 2020-05-21

**Authors:** Zhan-Miao Yi, Xu-Li Zhong, Ming-Lu Wang, Yuan Zhang, Suo-Di Zhai

**Affiliations:** ^1^Department of Pharmacy, Peking University Third Hospital, Beijing, China; ^2^Department of Pharmacy Administration and Clinical Pharmacy, School of Pharmaceutical Science, Peking University Health Science Center, Beijing, China; ^3^Institute for Drug Evaluation, Peking University Health Science Center, Beijing, China; ^4^Department of Pharmacy, Children's Hospital of Capital Institute of Pediatrics, Beijing, China; ^5^Department of Pharmacy, Shengjing Hospital of China Medical University, Shenyang, China; ^6^Department of Health Research Methods, Evidence and Impact, McMaster University, Hamilton, ON, Canada

**Keywords:** seizure cessation, seizure freedom, mortality, artificial ventilation, agitation, hypotension, cost-effectiveness

## Abstract

**Objective:**

To evaluate efficacy, safety, and economics profiles of intravenous levetiracetam (LEV) for status epilepticus (SE).

**Methods:**

We searched PubMed, Embase, the Cochrane Library, Clinicaltrials.gov, and OpenGrey.eu for eligible studies published from inception to June 12^th^ 2019. Meta-analyses were conducted using random-effect model to calculate odds ratio (OR) of included randomized controlled trials (RCTs) with RevMan 5.3 software.

**Results:**

A total of 478 studies were obtained. Five systematic reviews (SRs)/meta-analyses, 9 RCTs, 1 non-randomized trial, and 27 case series/reports and 1 economic study met the inclusion criteria. Five SRs indicated no statistically significant difference in rates of seizure cessation when LEV was compared with lorazepam (LOR), phenytoin (PHT), or valproate (VPA). Pooled results of included RCTs indicated no statistically significant difference in seizure cessation when LEV was compared with LOR [OR = 1.04, 95% confidence interval (CI) 0.37 to 2.92], PHT (OR = 0.90, 95% CI 0.64 to 1.27), and VPA (OR = 1.47, 95% CI 0.81 to 2.67); and no statistically significant difference in seizure freedom within 24 h compared with LOR [OR = 1.83, 95% CI 0.57 to 5.90] and PHT (OR = 1.08, 95% CI 0.63 to 1.87). Meanwhile, LEV did not increase the risk of mortality during hospitalization compared with LOR (OR = 1.03, 95% CI 0.31 to 3.39), PHT (OR = 0.89, 95% CI 0.37 to 2.10), VPA (OR = 1.28, 95% CI 0.32 to 5.07), and placebo (plus clonazepam, OR = 0.73, 95% CI 0.16 to 3.38). LEV had lower need for artificial ventilation (OR = 0.23, 95% CI 0.06 to 0.92) and a lower risk of hypotension (OR = 0.15, 95% CI 0.03 to 0.84) compared to LOR. A trend of lower risk of hypotension and higher risk of agitation was found when LEV was compared with PHT. Case series and case report studies indicated psychiatric and behavioral adverse events of LEV. Cost-effectiveness evaluations indicated LEV as the most cost-effective non-benzodiazepines anti-epileptic drug (AED).

**Conclusions:**

LEV has a similar efficacy as LOR, PHT, and VPA for SE, but a lower need for ventilator assistance and risk of hypotension, thus can be used as a second-line treatment for SE. However, more well-conducted studies to confirm the role of intravenous LEV for SE are still needed.

## Introduction

Status epilepticus (SE) is a relatively common and life-threatening neurological emergency with an estimated incidence of up to 61 episodes per 100,000 population per year (Betjemann and Lowenstein, 2015; Trinka and Kalviainen, 2017). The overall mortality rate of refractory SE is 17% to 27%, and up to 36% in super-refractory SE (Brophy et al., 2012; Kantanen et al., 2015). Predictably, its substantial morbidity and mortality could incur high health care costs. The total direct and indirect costs for epilepsy in Europe would add up to €13.8 billion per year, of which SE was an important source of direct costs (Olesen et al., 2012). In Germany, the average cost of hospitalization for SE was €14,946 per patient per admission with a mean length of stay was 19 days, and super-refractory SE (SRSE) was the main cost-drive with the estimated cost up to €50,488 (Kortland et al., 2016). The total cost of direct hospitalization in the United States (US) caused by SE each year was even as high as $4 billion (Penberthy et al., 2005). The International League against Epilepsy (ILAE)'s updated definition of SE in 2015 highlights the long-term effects of prolonged seizure activity on the nervous system, including neuronal death and neuronal injury (Trinka et al., 2015a). Therefore, the key of SE treatment strategy is to identify and terminate seizure activities as early as possible before irreversible neuronal damage occurs (Trinka and Kalviainen, 2017).

Currently, there is high level evidence only for the first-line medications of SE including intravenous (IV) benzodiapines (preferably lorazepam, LOR) (Shorvon et al., 2008; Meierkord et al., 2010; Capovilla et al., 2013; Glauser et al., 2016). But first-line therapy may fail to control at least 30%–40% of the time and LOR could not be obtained in some countries or areas, alternative treatment is necessary (Alldredge et al., 2001; Trinka et al., 2015b). Some of the conventional agents including phenytoin (PHT), phenobarbital (PB), and valproate (VPA) were available as second-line treatment. However, toxicity limited the applications, such as hypotension and respiratory suppression in PB, and hepatic injury in VPA (Sanchez Fernandez and Loddenkemper, 2015; Trinka et al., 2016). Therefore, newer, more effective and less toxic drugs for management of SE were needed. More recently, some new anti-epileptic drugs (AEDs) with IV dosage form, such as levetiracetam (LEV) and laconamide, have been used as alternative AEDs for the treatment of SE (Prasad et al., 2005; Trinka, 2011).

In 1999, LEV was approved as adjunctive therapy for adults with focal epilepsy in the US. In 2006, the US Food and Drug Administration approved IV LEV for patients above 16 years old when oral treatment was not feasible. Since then, efficacy of IV LEV has been reported in many open-label case-series in adults and children with SE. And some retrospective studies that have suggested LEV as an effective treatment for various forms of SE as a first- or second-, or third-line treatment (Berning et al., 2009; Moddel et al., 2009; Trinka and Dobesberger, 2009; Tripathi et al., 2010; Khan et al., 2011). Current researches suggested that LEV may bind to the synaptic vesicle protein 2A, and thus depressed the epilepsy discharge through participating in the exocytosis of synaptic vesicles and regulating the release of neurotransmitters (especially the excitatory amino acids) (Rigo et al., 2002; Matagne et al., 2008; Deshpande and Delorenzo, 2014). LEV has a favorable pharmacokinetic profile. No clinically significant interactions with other AEDs were found since LEV is mainly renal excreted unchanged independent of liver cytochrome P450 (Emswiler and Cumpston, 2017) Compared to many other AEDs, LEV had fewer reported adverse events (AEs), but this may be because of its relatively short period of clinical use (Trinka and Dobesberger, 2009; Trinka, 2011). In recent years, apart from psychiatric and behavioral side effects (Yi et al., 2018), some rare AEs of LEV including rhabdomyolysis, thrombocytopenia and anaphylaxis were reported (Koklu et al., 2014; Di Lorenzo and Li, 2017; Kim and Shin, 2017).

Thus, we conducted a systematic review (SR) and meta-analysis to evaluate the evidence on the efficacy, safety and economic benefits of IV LEV compared with all other AEDs for SE.

## Materials and Methods

### Search Strategy

We searched PubMed, Embase, Cochrane Library, ClinicalTrials.gov, and OpenGrey.eu from inception to July 19^th^ 2018 and updated the search results till June 12^th^, 2019. The following keywords were used in search terms for relevant literatures of LEV: “status epilepticus,” “epilepsy,” “seizures,” and “epileptic*” for the disease, and terms “Keppra,” “levetiracetam,” “Desitrend,” “Spritam,” “Kepcet,” “Kevtam,” “Levitam,” “injection,” and “intravenous” for the medication. We used the Boolean logic “AND” to combine the two sets of terms. The study was registered on PROSPERO (No. CRD 42017069367).

### Study Selection and Outcome Measures

Two independent authors (X-LZ and M-LW) screened all retrieved records for potentially eligible studies manually by title and abstract screening in the first stage, and the full-text screening secondly. During the title and abstract screening, studies that may meet the inclusion criteria, or need further information for a clear judgment were included for a full-text screening process. Studies were included if: (1) enrolled SE patients, (2) compared the efficacy, safety or economic profiles of IV LEV against all other anticonvulsants for SE, with no restriction to dosage and duration, and (3) SR, meta-analysis, randomized controlled trials (RCTs), observational studies (cohort and case–control studies, case reports, and case series) and economic studies were considered. Studies were excluded if: (1) original data was not presented (for example, comments and expert opinions), (2) published in non-English languages without required data translated into English, and (3) data extraction/interpretation was not possible. Disagreements was resolved through discussion, and a third party was consulted and discussed (Z-MY) if necessary.

The primary efficacy outcomes focused on SE cessation. The secondary efficacy outcomes included seizures freedom within 24 h, neurological outcome at discharge, need for ventilator assistance, and mortality during hospitalization, discontinuation due to AEs, serious AEs, total AEs, single AEs, and cost-effectiveness.

### Data Extraction and Quality Assessment

Two independent investigators (X-LZ and M-LW) performed data extraction according to a pre-designed data collection form. Information including authors, publication year, number of LEV trials, participant characteristics (participation-eligibility criteria, gender and age), dosage, treatment duration, outcomes of interest, and drop-out rate were extracted.

Methodological quality of included studies was independently assessed by the two investigators. The quality of included SRs was assessed with the Assessment of Multiple Systematic Reviews tool (range, 0~11) (Shea et al., 2007). The risk of bias in the eligible RCTs was assessed with the Cochrane risk of bias assessment tool (Higgins et al., 2011). The quality of the eligible economic study was assessed with consolidated health economic evaluation reporting standard (CHEERS) (Husereau et al., 2013) We did not conduct a quality assessment of the included observational studies. The authors of eligible studies were contacted for clarifications if there was missing data. We resolved all disagreements about data extraction and quality assessment through discussion among all authors.

### Statistical Analysis

Treatment effect of RCTs evaluating similar interventions in similar participants was pooled using random-effect model through meta-analyses in an intention to treat manner (following the allocation of participants in studies) with RevMan 5.3 software. The odds ratio (OR) for categorical outcomes was calculated. *P* < 0.05 was considered statistically significant. Safety outcomes of observational studies were described and numbers of case reports as well as case-series was pooled by classification of AEs. Statistical heterogeneity was assessed with the Mantel-Haenszel chi-square test and quantified with the *I*^2^ test.

## Results

### Study Selection

**Figure 1:**
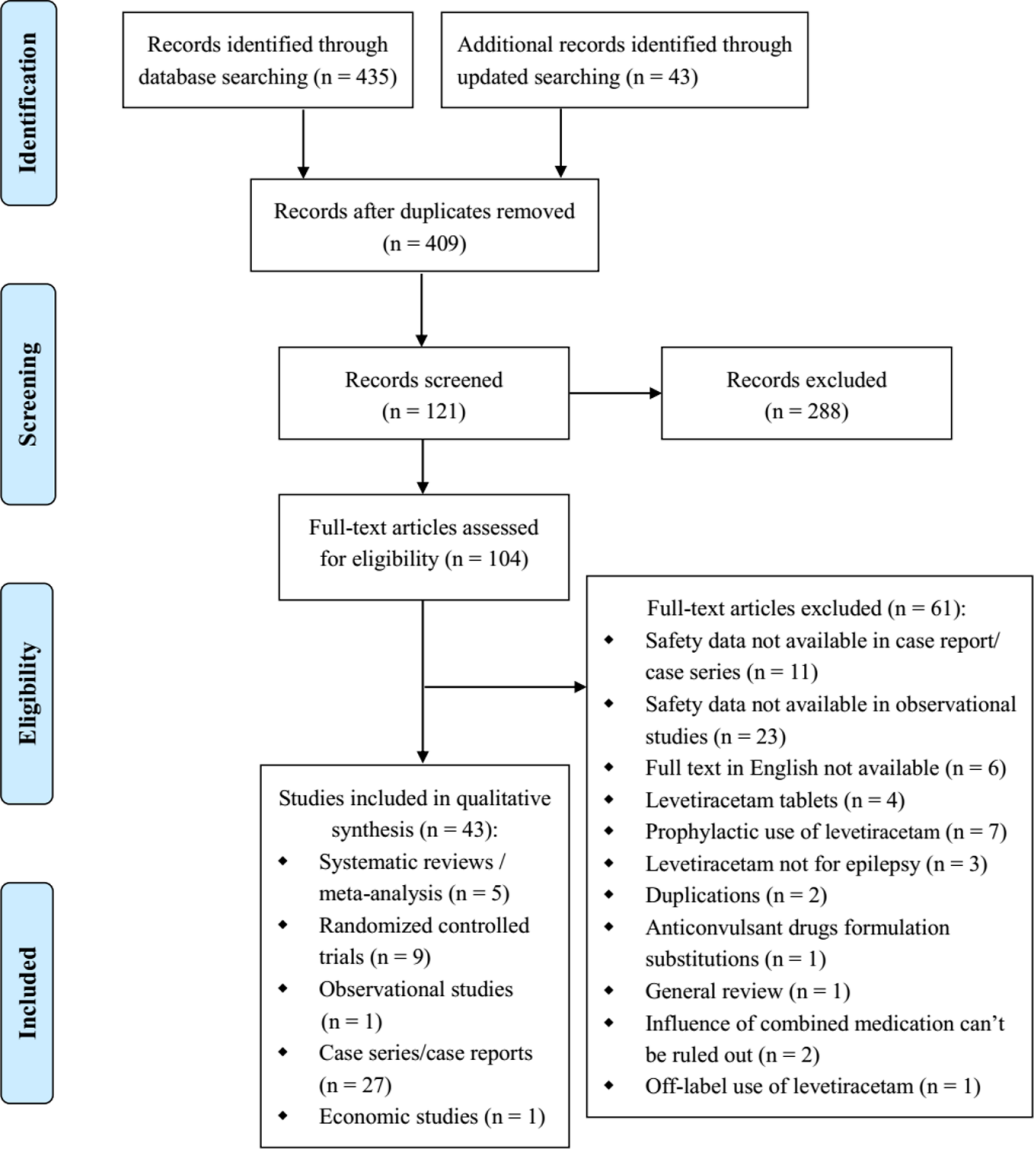
PRISMA flow diagram for literature search and study selection.

There were 435 relevant studies identified in the initial search, and 43 studies were identified in the updated search. After duplicates removed, we screened 409 title and abstract records. Of these, 288 studies were excluded after title/abstract screening, and 104 studies were selected for full-text review. And 43 reports met the inclusion criteria after full-text review: 5 SRs/meta-analyses (Liu et al., 2012; Zelano and Kumlien, 2012; Prasad et al., 2014; Yasiry and Shorvon, 2014; Brigo et al., 2016), 9 RCTs (Misra et al., 2012; Chakravarthi et al., 2015; Mundlamuri et al., 2015; Navarro et al., 2016; Gujjar et al., 2017; Singh et al., 2018; Dalziel et al., 2019; Lyttle et al., 2019; Nene et al., 2019), 1 non randomized trial (Tripathi et al., 2010), 27 case series/reports (Falip et al., 2006; Baulac et al., 2007; Michaelides et al., 2008; Ruegg et al., 2008; Abend et al., 2009; Berning et al., 2009; Beyenburg et al., 2009; Kirmani et al., 2009; Moddel et al., 2009; Wheless et al., 2009; Depositario-Cabacar et al., 2010; Ng et al., 2010; Reiter et al., 2010; Usery et al., 2010; Khan et al., 2011; Standish et al., 2011; Bahr et al., 2012; McTague et al., 2012; Rakshasbhuvankar et al., 2013; Isguder et al., 2014; Koklu et al., 2014; Atmaca et al., 2015; Lang et al., 2015; Bernatowicz and Li, 2017; Kim and Shin, 2017; May et al., 2017; Shahbaz et al., 2017), and 1 economic study (Sánchez Fernández et al., 2019) (**Figure 1**).

### Study Characteristics and Quality Assessment

The included SRs published between 2012 and 2016, enrolling patients with convulsive SE, non-convulsive SE and refractory SE. Two SRs compared LEV with VPA (Liu et al., 2012; Brigo et al., 2016), 1 SR compared LEV with PHT (Brigo et al., 2016), 1 SR compared LEV with LOR (Prasad et al., 2014) and 2 SRs evaluated the efficacy of LEV as first or second-line therapy in SE (Zelano and Kumlien, 2012; Yasiry and Shorvon, 2014) Among the two SRs, one SR evaluated the efficacy of five AEDs (included LEV) as a second-line therapy in benzodiazepine-resistant SE (Yasiry and Shorvon, 2014), another one evaluated LEV as a first-line AED, as single treatment or combined with benzodiazepines (Zelano and Kumlien, 2012). Characteristics of included SRs were showed in **Table 1**.

**Table 1:** The characteristics of included systematic reviews.

Author, year	Last search time	Patients	Studies	Cases	Intervention group	Control group	Outcome measures
Brigo et al. (2016)	2015.10	Convulsive SE (generalized or focal) persisting despite first-line BZDs in patients of any age	2	194	LEV	PHT/VPA	Number of patients with SE cessation within 15 min after the start of drug administration
Liu et al. (2012)	2010.11	All patients above 14 years old with refractory SE	1	82	LEV	VPA	SE cessation
Prasad et al. (2014)	2013.08	Premonitory or early stage SE or established SE	1	79	LEV	LOR	SE cessation; seizure recurrence within 24 h; hypotension; respiratory failure; death; ventilatory requirement
Yasiry and Shorvon (2014)	Not reported	Benzodiazepine-resistant convulsive SE	8	204	LEV	Not reported	SE cessation
Zelano and Kumlien (2012)	2011.01	Adults with SE	10	334	LEV combined with benzodiazepine	LEV alone	SE cessation; adverse events

BZDs, benzodiazepines; LEV, levetiracetam; LOR, lorazepam; PHT, phenytoin; SE, status epilepticus; VPA, valproate.

Among the included RCTs, 6 compared LEV with PHT for second-line treatment (Chakravarthi et al., 2015; Mundlamuri et al., 2015; Gujjar et al., 2017; Singh et al., 2018; Dalziel et al., 2019; Lyttle et al., 2019), 2 compared LEV with VPA (Mundlamuri et al., 2015; Nene et al., 2019), 1 compared LEV with LOR (Misra et al., 2012), and 1 compared LEV plus clonazepam with placebo plus clonazepam (Navarro et al., 2016), One non randomized trial compared LEV with VPA was included (Tripathi et al., 2010). Study characteristics of included RCTs and the non randomized trial were showed in **Table 2**.

**Table 2:** The characteristics of included randomized controlled trials and the observational study

Author, year	Basic characteristics of patients			Intervention		Evaluation period	Follow-up	Drop-out	Outcome measures
	Patients	Sample size (M/F)	Age (Mean ± SD)	Intervention group	Control group				
Chakravarthi et al. (2015)	Aged 14~75 years old with SE uncontrolled with LOR	44 (27/17)	35.41 ± 16.03	IV LEV: 20 mg/kg (rate 100 mg/min) followed by maintenance doses	IV PHT: 20 mg/kg (maximum rate 50 mg/min) followed by maintenance doses	24 h	Follow-up to discharge	None	① Primary: termination of seizure activity within 30 min ② Secondary: recurrence of seizures within 24 h, drug related AEs, neurological outcome, need for ventilatory assistance, mortality during hospitalization
Gujjar et al. (2017)	aged above 15 years old with SE	52 (34/18)	37.8 ± 18	IV LEV: 30 mg/kg over 30 min followed by maintenance doses	IV PHT: 20 mg/kg within 30 min followed by maintenance dose	24h	Follow-up to discharge	None	① Primary: control of seizure with no recurrence over next 24 h. ② Secondary: AEs
Misra et al. (2012)	Convulsive SE	79 (51/28)	LEV group: 39.16 ± 21.16 LOR group: 38.90 ± 23.25	IV LEV: 20 mg/kg infused in 15 min	IV LOR: 0.1 mg/kg in 10 ml saline IV in 2–4 min	24 h	Follow-up to discharge	4*	① Primary: clinical seizure cessation within 30 min ② Secondary: 24-h seizure freedom, mortality, AEs
Mundlamuri et al. (2015)	Aged 15~65 years old with GCSE	150 (88/62)	33.71 ± 17.00	IV LEV: 25 mg/kg over 15 minutes followed by maintenance dose	IV PHT: 20 mg/kg followed by maintenance dose IV VPA: 30 mg/kg followed by maintenance dose	24h	1 month	1st line treatment: none.	① Primary: seizure cessation of first AED; ② Secondary: medical complications, mortality, major AEs, functional good outcome
Navarro et al. (2016)	Aged above 18 years old with GCSE	136 (94/42)	LEV group: 55 ± 18 Placebo group 53 ± 18	IV CZP + IV LEV: 1 mg CZP for 1 min with 2~5 g LEV for 5 min	IV CZP + IV Placebo: 1 mg CZP for 1 min with placebo for 5 min	35min	15 days	Death: 7 cases^†^ Unexplained exfoliation: 5 cases^‡^	① Primary: cessation of convulsions within 15 min ② Secondary: No need of ventilatory assistance, mortality during hospitalization
Dalziel et al. (2019)	Aged between 3 months and 16 years old with convulsive SE that failed first-line BZDs	233 (112/121)	3.9 ± 3.8 LEV group: 3.8 ± 3.8 PHT group: 4.0 ± 3.9	IV LEV: 40 mg/kg intravenous or intraosseous LEV infusion over 5 min	IV PHT: 20 mg/kg intravenous or intraosseous PHT infusion over 20 min	24 h	1 month and 2 months	1 (PHT group, died)	① Primary: clinical cessation of seizure activity 5 min ② Secondary: clinical cessation of seizure activity 2 h, serious AEs
Lyttle et al. (2019)	Aged 6 months and 18 years old with convulsive SE that required second-line treatment	286 (147/139)	Median (IQR) LEV group: 2.7 (1.3 ~ 5.9) PHT group: 2.7 (1.6 ~ 5.6)	IV LEV: 40 mg/kg over 5 min, maximum dose 2.5 g	IV PHT: 20 mg/kg over at least 20 min, maximum dose 2 g and with a maximum infusion rate of 1 mg/kg/min	24 h	14 days	5^#^	① Primary: time randomization to cessation of all visible signs of convulsive activity ② Secondary: need for further anticonvulsants after the trial treatment; serious AEs
Nene et al. (2019)	Aged above 60 years old with GCSE	118 (73/45)	Overall: 67.5 ± 7.5 LEV group: 66.6 ± 6.7 VPA group: 68.5 ± 8.0	IV LEV: 20–25 mg/kg over 15 min followed by maintenance dose	IV VPA: 20–25 mg/kg followed by maintenance dose	24 ~ 48 h	1 month	18	① Primary: response to first line AEDs. ② Secondary: thirty-day mortality.
Singh et al. (2018)	Aged 3 to 12 years old with focal motor seizures and second episode of generalized seizures	100 (58/42)	Overall: NA LEV group: 7.35 ± 2.20 PHT group: 7.03 ± 2.85	IV LEV: 30 mg/kg at 5 mg/kg/min with maintenance dose	IV PHT: 20 mg/kg at 1 mg/kg/min with maintenance dose	24 h	7 days	5 (3 in LEV, 2 PHT)	① Primary: absence of seizure activity within next 24 h. ② Secondary: changes in respiratory rate, heart rate, blood pressure, and oxygen saturation at various time points, achievement of therapeutic drug levels
Tripathi et al. (2010)	Aged above 14 years old with refractory SE.	82 (42/40)	LEV group: 21.08 ± 9.70 VPA group: 26.62 ± 10.10	IV LEV: 30 mg/kg	IV VPA: 30 mg/kg	24 h	NA	NA	① Primary: termination of seizure activity within 30 min ② Secondary: seizures freedom within 24 h

*A total of 83 patients with SE were recruited in this study, 4 were excluded because of lack of consent, liver failure, already on study drug, and nonconvulsive SE in 1 each.

^†^Three cases died in LEV group and four cases died in placebo group.

^‡^After 15 days of follow-up, there were five cases of unexplained exfoliation: two cases in LEV group and three cases in placebo group.

^#^Two participants in the PHT group discontinued treatment early because of loss of intravenous access during drug administration, one participant died in LEV group and one participant died in PHT group, and one participant died as a result of catastrophic cerebral edema unrelated to either treatment (this participant received LEV followed by PHT)

AE, adverse events; AED, anti-epileptic drugs; BZDs, benzodiazepines; F, female; GCSE, generalized convulsions status epilepticus; IQR, interquartile range; IV, intravenous; LEV, levetiracetam; LOR, lorazepam; M, male; NA, not available; PHT, phenytoin; SD, standard difference; SE, status epilepticus; VPA, valproate.

Study characteristics of included 27 case reports/series were showed in **Table 3**.

**Table 3:** The characteristics of included case reports/series.

Psychiatric and behavioral side effects (n = 15)	Digestive system (n = 7)	Hematological side effects (n = 3)	Kidney (n = 2)	Skin (n = 2)	Seizure aggravation (n = 2)	Others (n = 11)
Atmaca et al. (2015)	Berning et al. (2009)	Kim and Shin (2017)	Abend et al. (2009)	Kirmani et al. (2009)	Depositario-Cabacar et al. (2010)	Bahr et al. (2012)
Bahr et al. (2012)	Lang et al. (2015)	Michaelides et al. (2008)	Baulac et al. (2007)	Wheless et al. (2009)	Ng et al. (2010)	Baulac et al. (2007)
Baulac et al. (2007)	Moddel et al. (2009)	Ruegg et al. (2008)				Bernatowicz and Li (2017)
Beyenburg et al. (2009)	Reiter et al. (2010)					Lang et al. (2015)
Falip et al. (2006)	Usery et al. (2010)					Koklu et al. (2014)
Isguder et al. (2014)	Standish et al. (2011)					May et al. (2017)
Khan et al. (2011)	Rakshasbhuvankar et al. (2013)					Michaelides et al. (2008)
Kirmani et al. (2009)						Ng et al. (2010)
Lang et al. (2015)						Reiter et al. (2010)
McTague et al. (2012)						Wheless et al. (2009)
Michaelides et al. (2008)						Shahbaz et al. (2017)
Ng et al. (2010)						
Reiter et al. (2010)						
Standish et al. (2011)						
Usery et al. (2010)						

A total of one economic study was from the U.S. evaluating the cost-effectiveness of non-benzodiazepine AEDs (non-BZD AEDs) for treatment of BZD-resistant convulsive SE was included (Sánchez Fernández et al., 2019). Decision analysis model populated with effectiveness data from a SR and meta-analysis of literature, and cost data from publicly available prices. The primary outcome was cost per seizure stopped.

Generally, included SRs and economic study was of good quality, but RCTs were of unclear or high risk of bias. Of the five included SRs, two SRs were of high quality (Prasad et al., 2014; Brigo et al., 2016), two SRs were of moderate quality due to unclear information in 3 items and unreported information in 2 items in Liu et al. (2012) while unclear information in 6 items and unreported information in 2 items in Yasiry 2014 (Yasiry and Shorvon, 2014), and one SR was of low quality due to unclear or unreported information in 10 of 11 items (Zelano and Kumlien, 2012). The included nine RCTs were generally of unclear or high risk of bias mainly due to unclear risk of bias in allocation concealment and blinding domains, only two RCTs were of low risk of bias (Dalziel et al., 2019; Lyttle et al., 2019). The included RCTs were generally open-label randomized clinical trials, only one RCT used the double-blind design (Navarro et al., 2016) and one RCT used the single-blind design (Nene et al., 2019). All included nine RCTs analyzed data with the intention-to-treat principle (**Table 4**). The included economic study was of high quality, as it covered most of the items specified by the CHEERS statement except descriptions of study perspective and discount rate (Sánchez Fernández et al., 2019).

**Table 4:** Risk of bias of included randomized controlled trials.

Studies	Random sequence generation	Allocation concealment	Blinding	Incomplete outcome data	Selecting reporting	Other source of bias	Total
Chakravarthi et al. (2015)	High	High	Unclear	Low	Low	Low	High
Gujjar et al. (2017)	Low	Unclear	Unclear	Low	Low	Low	Unclear
Misra et al. (2012)	Low	Unclear	Unclear	High	Low	Unclear	High
Mundlamuri et al. (2015)	Low	Unclear	Unclear	Low	Low	Low	Unclear
Navarro et al. (2016)	Low	Low	Low	Low	Low	High	High
Dalziel et al. (2019)	Low	Low	Low	Low	Low	Low	Low
Lyttle et al. (2019)	Low	Low	Low	Low	Low	Low	Low
Nene et al. (2019)	Low	Unclear	Unclear	Unclear	Low	Low	Unclear
Singh et al. (2018)	Low	Low	Unclear	Unclear	Low	Low	Unclear

### SE Cessation

Five SRs evaluated rates of SE cessation (Liu et al., 2012; Zelano and Kumlien, 2012; Prasad et al., 2014; Yasiry and Shorvon, 2014; Brigo et al., 2016). One SR indicated no statistically significant difference when LEV was compared with LOR (Prasad et al., 2014) as first-line treatment (risk ratio, RR= 0.97, 95% CI 0.44 to 2.13) or PHT (Brigo et al., 2016) as second-line treatment (OR = 1.18, 95% CI 0.50 to 2.79). Two SRs (Liu et al., 2012; Brigo et al., 2016) indicated no statistically significant difference when LEV was compared with VPA as second-line treatment (OR = 1.16, 95% CI 0.45 to 2.97, indirect comparisons; (Brigo et al., 2016) and OR = 0.80, 95% CI 0.32 to 2.01) (Liu et al., 2012). One SR indicated that the efficacy of LEV ranged from 44% to 94% (Zelano and Kumlien, 2012).

**Figure 2:**
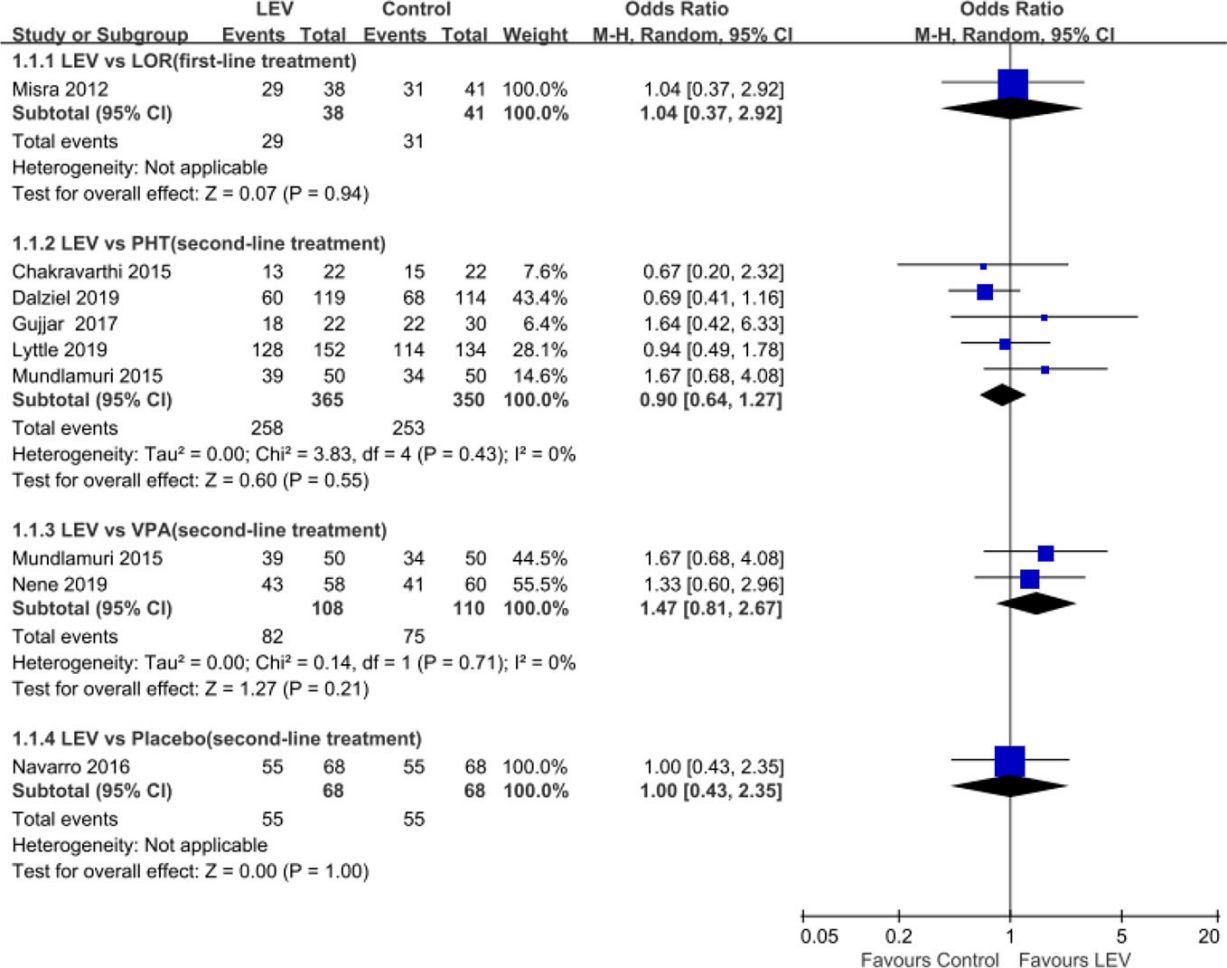
Rates of seizure cessation of included randomized controlled trials. Abbreviations: CLO, clonazepam; LEV, levetiracetam; LOR, lorazepam; PHT, phenytoin; VPA, valproate.

Meta-analyses of newly included RCTs showed no statistically significant difference in SE cessation when LEV was compared with LOR (Misra et al., 2012) (first-line treatment, OR = 1.04, 95% CI 0.37 to 2.92), PHT (Chakravarthi et al., 2015; Mundlamuri et al., 2015; Gujjar et al., 2017; Singh et al., 2018; Dalziel et al., 2019; Lyttle et al., 2019) (second-line treatment, OR = 0.90, 95% CI 0.64 to 1.27), and VPA (Mundlamuri et al., 2015; Nene et al., 2019) (second-line treatment, OR = 1.47, 95% CI 0.81 to 2.67). And there was no statistically significant difference when LEV plus clonazepam was compared with placebo plus clonazepam (Misra et al., 2012) (OR = 1.00, 95% CI 0.43 to 2.35) (**Figure 2**).

### Seizure Freedom Within 24 h

**Figure 3:**
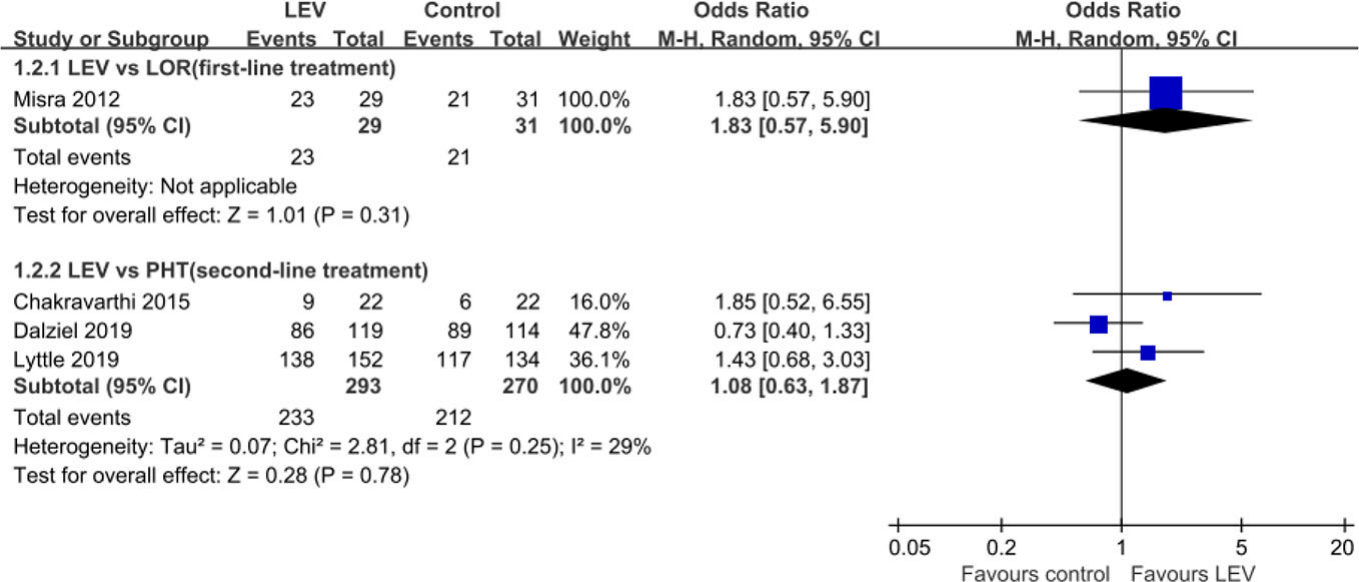
Number of seizure freedom within 24 h of included randomized controlled trials.

Four RCTs (Misra et al., 2012; Chakravarthi et al., 2015; Dalziel et al., 2019; Lyttle et al., 2019) evaluated the rates of seizure freedom within 24 h and indicated no statistically significant difference in rates of seizure freedom within 24 h when LEV was compared with LOR (Misra et al., 2012) [first-line treatment, OR = 1.83, 95% confidence interval (CI) 0.57 to 5.90], PHT (Chakravarthi et al., 2015; Dalziel et al., 2019; Lyttle et al., 2019) (second-line treatment, OR = 1.08, 95% CI 0.63 to 1.87) (**Figure 3**).

### Neurological Outcome at Discharge

**Figure 4:**
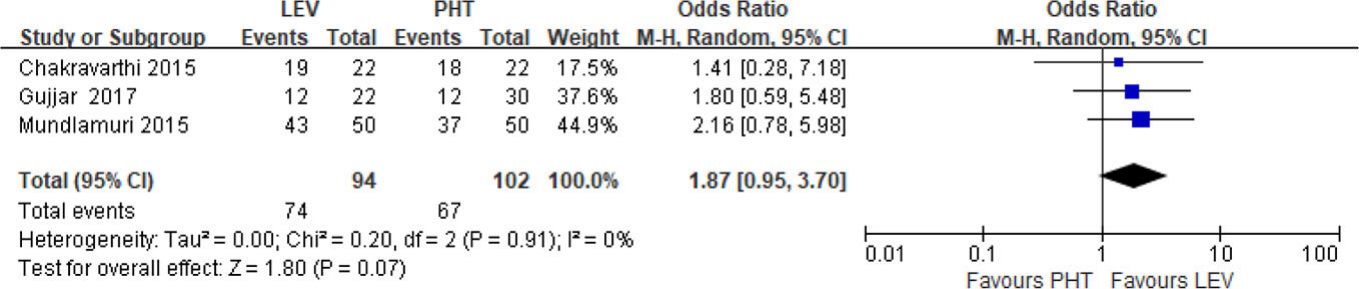
Number of functional good outcome at discharge of included randomized controlled trials.

Three RCTs (Chakravarthi et al., 2015; Mundlamuri et al., 2015; Gujjar et al., 2017) reported the neurological outcome at discharge. The definition of good functional outcome in one RCT is 5~7 points in functional independence measure (Chakravarthi et al., 2015) and 0~3 points in the modified Rankin scale in two RCTs (Mundlamuri et al., 2015; Gujjar et al., 2017). Pooled results showed no statistically significant difference between LEV and PHT in the number of good functional outcomes at discharge (OR = 1.87, 95% CI 0.95 to 3.70) (**Figure 4**).

### Mortality During Hospitalization

**Figure 5:**
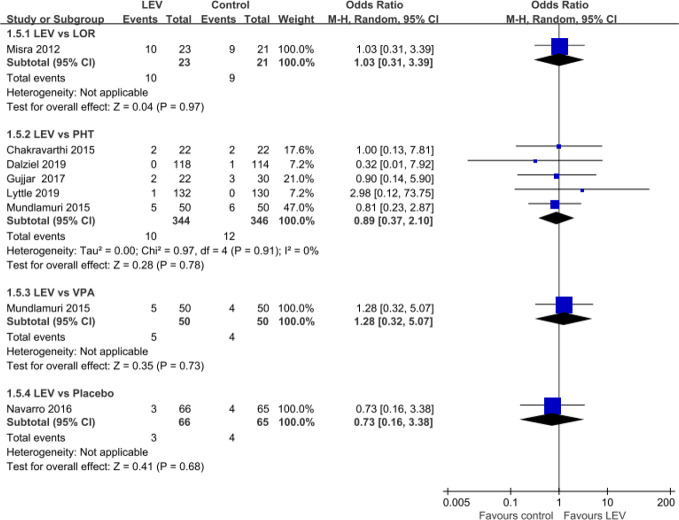
Rates of mortality during hospitalization of included randomized controlled trials.

Seven RCTs (Misra et al., 2012; Chakravarthi et al., 2015; Mundlamuri et al., 2015; Navarro et al., 2016; Gujjar et al., 2017; Dalziel et al., 2019; Lyttle et al., 2019) reported mortality during hospitalization. Pooled results showed no statistically significant difference in rates of mortality during hospitalization when LEV was compared with LOR (first-line treatment, OR = 1.03, 95% CI 0.31 to 3.39), PHT (second-line treatment, OR = 0.89, 95% CI 0.37 to 2.10), and VPA (second-line treatment, OR = 1.28, 95% CI 0.32 to 5.07), placebo (plus clonazepam, OR = 0.73, 95% CI 0.16 to 3.38) (**Figure 5**).

### Discontinuation Due to Adverse Events and Serious Adverse Events

The newly included RCTs showed no cases of discontinuation due to AEs.

What's more, the meta-analysis of newly included RCTs indicated that there was no statistically significant difference between LEV and PHT (OR = 0.65, 95% CI 0.11 to 3.96) in the rates of serious AEs (Dalziel et al., 2019; Lyttle et al., 2019), only one RCT reported serious AEs and indicated no statistically significant difference when LEV plus clonazepam was compared with placebo plus clonazepam (19/47 versus 28/47) (Navarro et al., 2016).

### Total Adverse Events

Among the five included SRs, only one SR (Zelano and Kumlien, 2012) reported AEs and indicated sedation/somnolence as the most common AEs with rates ranging from 12.5% to 40% with LEV.

**Figure 6:**
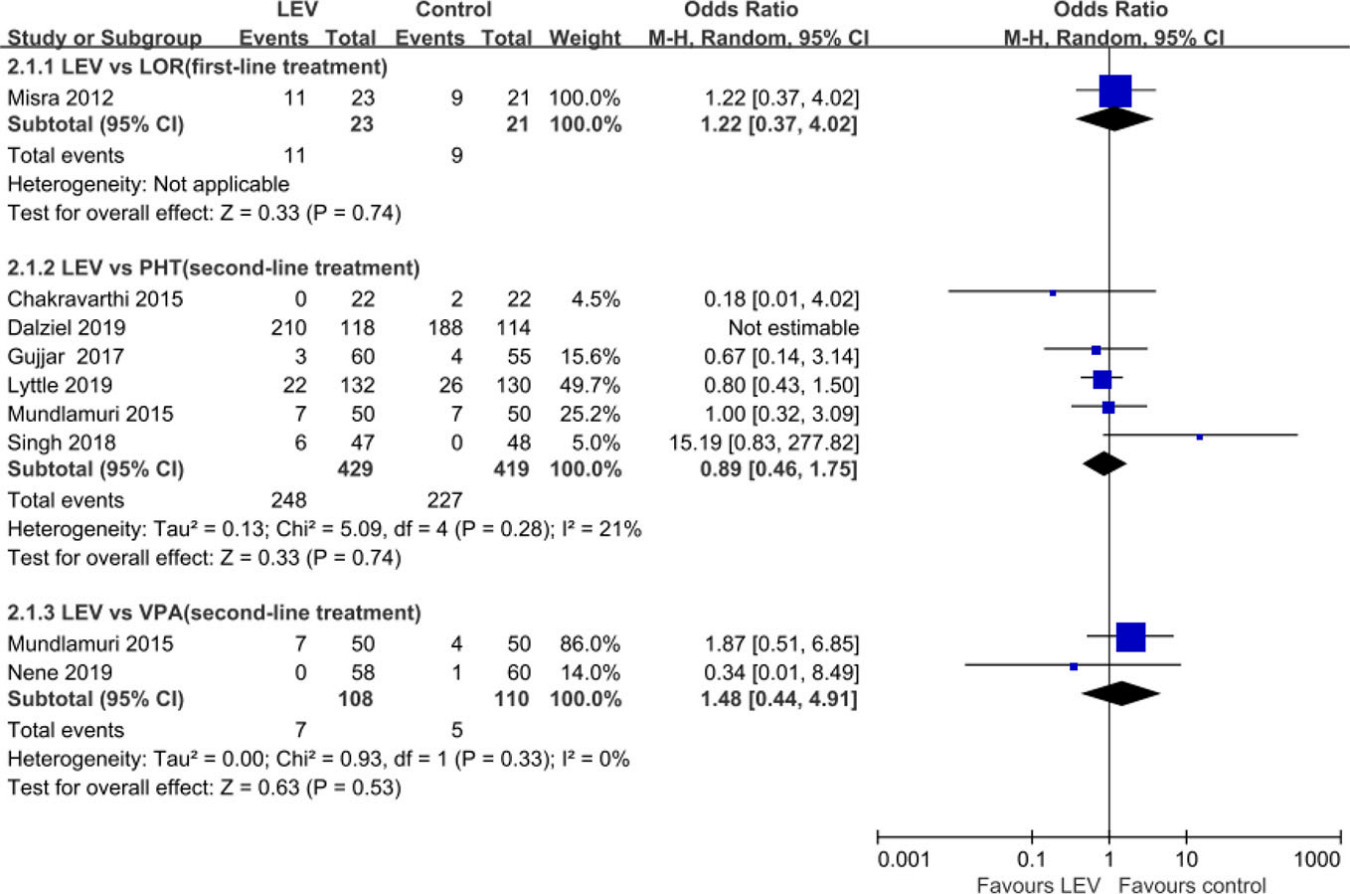
Total adverse events of included randomized controlled trials.

Nine RCTs (Misra et al., 2012; Chakravarthi et al., 2015; Mundlamuri et al., 2015; Navarro et al., 2016; Gujjar et al., 2017; Singh et al., 2018; Dalziel et al., 2019; Lyttle et al., 2019; Nene et al., 2019) reported total AEs. Meta-analysis of eight included RCTs (Misra et al., 2012; Chakravarthi et al., 2015; Mundlamuri et al., 2015; Gujjar et al., 2017; Singh et al., 2018; Dalziel et al., 2019; Lyttle et al., 2019; Nene et al., 2019) showed no statistically significant difference when LEV was compared with LOR (OR = 1.22, 95% CI 0.37 to 4.02) (Misra et al., 2012), PHT (OR = 0.89, 95% CI 0.46 to 1.75) (Chakravarthi et al., 2015; Mundlamuri et al., 2015; Gujjar et al., 2017; Singh et al., 2018; Dalziel et al., 2019; Lyttle et al., 2019), and VPA (OR = 1.48, 95% CI 0.44 to 4.91) (Mundlamuri et al., 2015; Nene et al., 2019) in rates of total AEs (**Figure 6**). Another RCT reported lower but no statistically significant difference total non-serious AEs of LEV plus clonazepam compared with placebo plus clonazepam (90/197 versus 107/197) (Navarro et al., 2016). None of AEs led to withdrawal from drug treatment.

### Single AEs

One RCT compared LEV plus clonazepam and placebo plus clonazepam study reported the most common AEs as respiratory disorders (32%), general disorders (19%), musculoskeletal and connective tissue disorders (12%), psychiatric disorders (12%), infections (10%), and nervous system disorders (9%) in LEV plus clonazepam group, while the most common AEs as respiratory disorders (32%), musculoskeletal and connective tissue disorders (19%), general disorders (18%), infections (16%), gastrointestinal disorders (15%), and vascular disorders (12%) in LEV plus placebo group (Navarro et al., 2016).

One RCT study indicated a lower need for artificial ventilation (4/23 vs 10/21, OR = 0.23, 95% CI 0.06~0.92) and a lower risk of hypotension (2/23 vs 8/21, OR = 0.15, 95% CI 0.03~0.84) when LEV compared with LOR. Other AEs in LEV and LOR groups were pneumonia (8/7), thrombocytopenia (4/1), agitation (4/0), liver dysfunction (0/2), rash (1/0), and urinary infection (0/1), with no statistic difference (Misra et al., 2012).

Six included RCTs (Chakravarthi et al., 2015; Mundlamuri et al., 2015; Gujjar et al., 2017; Singh et al., 2018; Dalziel et al., 2019; Lyttle et al., 2019) compared the incidence of single AEs for LEV and PHT. Four of these studies (Chakravarthi et al., 2015; Mundlamuri et al., 2015; Gujjar et al., 2017; Lyttle et al., 2019) indicated that LEV had a trend of lower risk of hypotension compared with PHT (OR = 0.34, 95% CI 0.10~1.21); while two included RCTs (Gujjar et al., 2017; Lyttle et al., 2019) indicated that LEV had a trend of higher risk of agitation (OR = 2.86, 95% CI 0.95~8.59).

One RCT indicated that LEV had a trend of higher risk of hallucinations compared with VPA (3/50 vs 0/50) (Mundlamuri et al., 2015). Another RCT indicated that LEV had a lower risk of hepatic dysfunction (0/58 vs 1/60) compared with VPA, but no difference was found (Nene et al., 2019). One non randomized trial showed that no AEs such as hepatic dysfunction, hypotension, abnormal behavior, depressed respiratory, or thrombocytopenia occurred in any group (Tripathi et al., 2010).

Among the 27 case reports/series, 15 reported psychiatric and behavioral AEs and 7 reported gastrointestinal AEs. Rates of behavioral AEs (such as aggression and irritability) varied from 1.1% to 15% and nausea/vomiting varied from 3.1% to 5.6% (**Table 2**).

### Cost-Effectiveness

One cost-effectiveness evaluation for BZD-resistant convulsive SE with decision analysis model was conducted in the US (Sánchez Fernández et al., 2019). The economic study showed the most cost-effective non-BZD AED was LEV (incremental cost-effectiveness ratio, incremental cost-effectiveness ratio, ICER: $18.55/seizure-stop), followed by VPA (ICER: $94.44/seizure-stop), and lastly PB (ICER: $847.22/seizure-stop). PHT and lacosamide were not cost-effective compared to the other options. Probabilistic sensitivity analysis indicated that LEV was the most cost-effective strategy in most second-order Monte Carlo simulations for a willingness to pay between approximately $25/seizure-stop and $100/seizure-stop.

## Discussion

The present study indicated that LEV had similar efficacy in SE cessation and mortality during hospitalization compared with LOR, PHT and VPA; no statistically significant difference in rates of seizure freedom within 24 h compared with LOR and PHT, as well as number of good functional outcome at discharge compared with PHT. Concerning safety, no statistically significant difference among LEV, LOR, PHT, and VPA in rates of total AEs was found. However, LEV had a lower risk of hypotension and requirement for ventilator assistance compared with LOR and PHT.

Available IV AEDs are limited in clinical practice. Considering AEs of AEDs, one medication may be preferable for patients. Hong Kong Epilepsy Guideline suggested that IV LEV can be considered as an alternative to PHT in benzodiazepine-resistant SE, such as established SE (Kellinghaus and Stogbauer, 2012; Fung and Fung, 2017; Lyttle et al., 2017). The National Institute for Health and Care Excellence guideline suggested that LEV are potentially as effective as PB and safer for SE (Lyttle et al., 2017). Compared with VPA, PHT and lacosamide, LEV was the first choice drug for IV administration of SE, according to a Germany chart review (Kellinghaus and Stogbauer, 2012).

In this study, we collected the most comprehensive evidence of IV LEV, while safety profiles were often missed in the previous studies (Zelano and Kumlien, 2012; Prasad et al., 2014; Yasiry and Shorvon, 2014; Chakravarthi et al., 2015). Second, types of literature included in the study include SRs, RCTs, and observational studies. The study can provide reliable evidence for physicians or policymakers.

There are still some limitations to this study. First, only English-language studies were included. Moreover, the included RCTs were of unclear or high risk of bias. SE needs rapid termination of seizure activity to minimize neurological injury and systemic dysfunction, so it is difficult to achieve strict and consistent randomized control. However, although most of the included RCTs were not double-blinded, the judgment of some outcomes, such as SE cessation was not influenced. Third, no subgroup analysis of different types of SE such as convulsive SE, non-convulsive forms of SE were conducted due to limited studies included.

Considering the complexity of SE and the relatively high incidence of complications, the management of SE and its pharmacological treatment still need more high quality evidence to inform clinical practice and decision making.

## Conclusions

IV LEV can be used as a second-line treatment for SE. For patients with SE, LEV has similar efficacy with LOR, PHT, and VPA, and a lower risk of hypotension and requirement for ventilator assistance compared with LOR and PHT. Regarding tolerability, LEV also showed good qualities for it does not increase risks of serious AEs or discontinuation from studies due to AEs in current researches. However, there is still a lack of evidence to support its cost-effectiveness, and more studies are needed to confirm its role for SE.

## Author Contributions

Z-MY conceived this review. X-LZ and M-LW identified reports of trials and extracted data. YZ provided statistical advice. Z-MY, X-LZ, and M-LW did all statistical analyses, checked for statistical inconsistency, and interpreted data. S-DZ contributed to data interpretation. Z-MY drafted the report and all other authors (X-LZ, M-LW, YZ, and S-DZ) critically reviewed the article. All authors read and approved the final manuscript.

## Funding

The authors declare that this study received funding from Beijing Pharmaceutical Association and UCB China Inc. The funder was not involved in the study design; collection, analysis, and interpretation of data; the writing of this article; or the decision to submit it for publication.

## Conflict of Interest

The authors declare that the research was conducted in the absence of any commercial or financial relationships that could be constructed as a potential conflict of interest.
